# MicroRNA-18a prevents senescence of mesenchymal stem cells by targeting CTDSPL

**DOI:** 10.18632/aging.205642

**Published:** 2024-03-07

**Authors:** Bo Sun, Xian-Hui Meng, Yu-Min Li, Hao Lin, Zhong-Dang Xiao

**Affiliations:** 1 State Key Laboratory of Digital Medical Engineering, School of Biological Science and Medical Engineering, Southeast University, Nanjing 210096, China; 2 Department of Clinical Science and Research, Zhongda Hospital, School of Medicine, Southeast University, Nanjing 210009, China

**Keywords:** mesenchymal stem cells, miR-18a-5p, CTDSPL, senescence

## Abstract

Stem cell therapy requires massive-scale homogeneous stem cells under strict qualification control. However, Prolonged *ex vivo* expansion impairs the biological functions and results in senescence of mesenchymal stem cells (MSCs). We investigated the function of CTDSPL in the premature senescence process of MSCs and clarified that miR-18a-5p played a prominent role in preventing senescence of long-term cultured MSCs and promoting the self-renewal ability of MSCs. Over-expression of CTDSPL resulted in an enlarged morphology, up-regulation of p16 and accumulation of SA-β-gal of MSCs. The reduced phosphorylated RB suggested cell cycle arrest of MSCs. All these results implied that CTDSPL induced premature senescence of MSCs. We further demonstrated that miR-18a-5p was a putative regulator of CTDSPL by luciferase reporter assay. Inhibition of miR-18a-5p promoted the expression of CTDSPL and induced premature senescence of MSCs. Continuous overexpression of miR-18a-5p improved self-renewal of MSCs by reducing ROS level, increased expression of Oct4 and Nanog, and promoted growth rate and differentiation capability. We reported for the first time that the dynamic interaction of miR-18a-5p and CTDSPL is crucial for stem cell senescence.

## INTRODUCTION

Stem cell therapy has been taken as a promising strategy for many tough diseases such as cardiac impairment [1], neurodegenerative disorders [2], systemic lupus erythematosus [3], etc. Mesenchymal stem cells (MSCs) exhibit multi-lineage differentiation potentials and attractive immune modification properties [4], in combination with their convenience of isolation and low possibility of tumorigenesis making them an ideal source for clinical usage [5]. However, considering their heterogeneity and the quality of the cell after an *ex vivo* expansion, the beneficial effect of long-term cultured MSCs is hard to be evaluated. Thus, the Maintenance of MSC properties during long-term culture without spontaneous differentiation or premature senescence is a great challenge for their further usage. It is well known that long-term cultured MSCs underwent considerable epigenetic and genetic changes [6–8], nevertheless, pivotal mechanisms that govern MSC’s self-renewal and senescence need to be further clarified.

MicroRNAs (miRNAs), as a kind of 18–24 nucleotides non-coding RNAs, modulate biological activities at the post-transcriptional level [9]. Previous studies demonstrated that they play essential roles at determining cell fate [10]. For example, miR-302 and miR-307 were demonstrated to regulate self-renewal of stem cells [11, 12]. On the other side, Senescence is always accompanied by aberrant expression of some specific miRNAs, such as miR-34a, miR-217, miR-335, miR-377, which were referred to as the senescence-associated miRNAs [13, 14].

In our previous study, we investigated the miRNA profile changes upon prolonged culture of human umbilical cord and umbilical cord blood-derived MSCs [15]. We found that long-term culture significantly up-regulated miR-26 family expression and down-regulated miR-17/92 cluster expression in MSCs. Studies on the miR-26 family have been widely associated with tumorigenesis [16]. Interestingly, the genomic loci of miR-26a and miR-26b localize to the introns of genes coding carboxy-terminal domain RNA polymerase II polypeptide A small phosphatase (CTDSP) family (CTDSP1, CTDSP2, CTDSPL) and a functional association between them has been well demonstrated by another study [17]. As a CTDSP family member, CTDSPL was previously discovered as a tumor suppressor gene in epithelial malignancies [18]. It works as a phosphatase involved in the regulation of cell growth, snail stability and TGF-β pathway activity [18, 19]. These studies suggest that CTDSPL potentially regulates cellular senescence, which has not been elaborated yet.

In this work, we examined the expression level of CTDSPL in cultured UCMSCs and showed that the up-regulation of CTDSPL significantly induced premature senescence of MSCs. We also found that miR-18a-5p inhibited CTDSPL function by direct targeting. Finally, abundant expression of miR-18a-5p played a prominent role in preventing senescence of long-term cultured MSCs and promoting the self-renew ability of MSCs.

## RESULTS

### Senescence impaired the biological functions of mesenchymal stem cells

**Figure 1:**
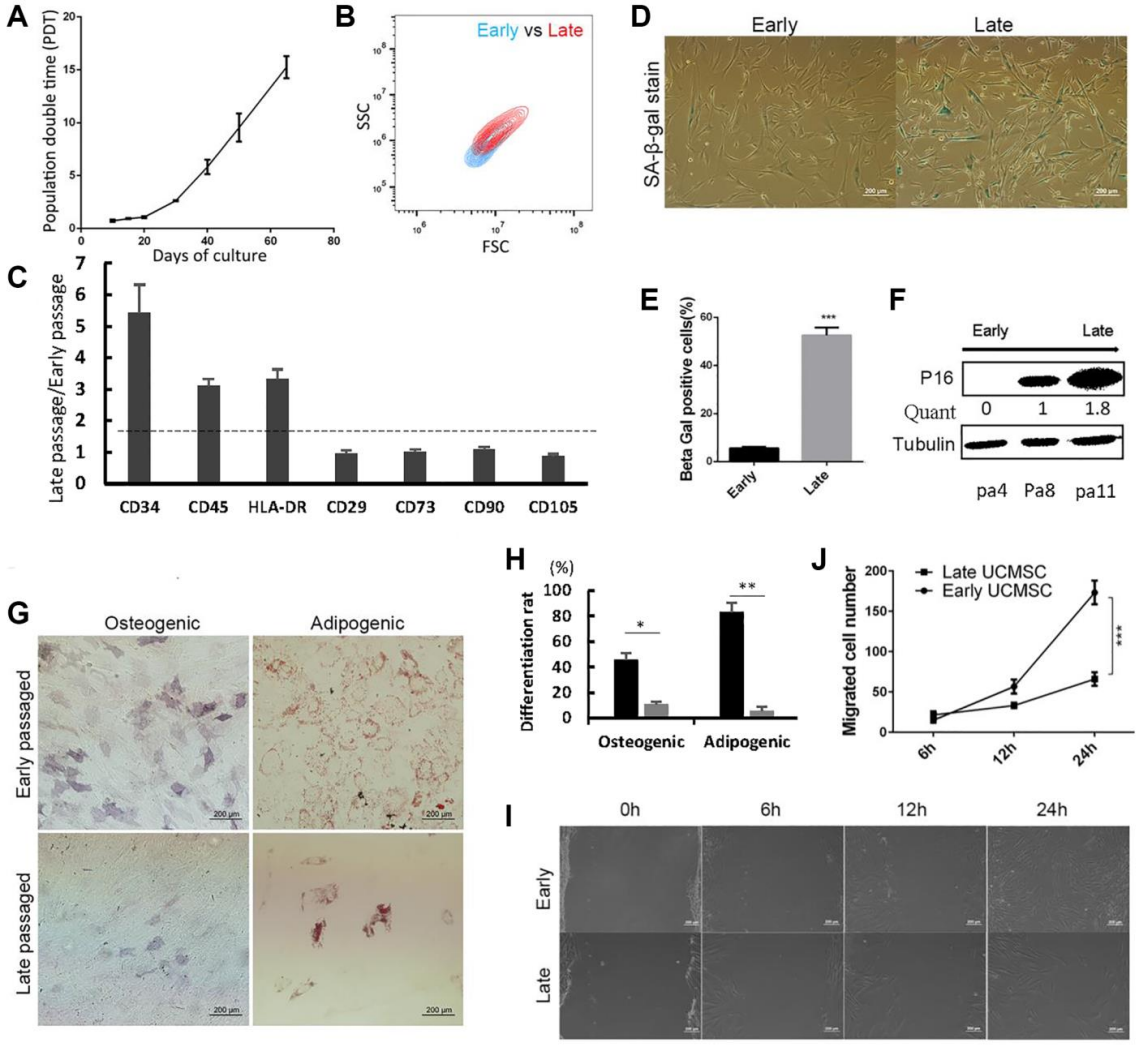
**Late passaged UCMSCs showed senescence-associated changes and impaired biological functions.** (**A**) Population double time (PDT) of UCMSCs during long-term expansion. (**B**) The cell size of early and late passaged UCMSCs was measured using forward scatter (FSC) and side scatter (SSC) by flow cytometry. (**C**) Immunophenotypes characterization of early and late passaged UCMSCs. (**D**) SA-β-gal staining of early and late passaged UCMSCs. (**E**) Quantification of the SA-β-gal staining positive cells (*n* = 3). (**F**) Western blot analysis of p16 expression in UCMSCs of passages 4, 8, and 11. (**G**) Early and late passaged UCMSCs were induced to osteogenic (Ost) or adipogenic (Ado) differentiation. Osteogenic efficiency was evaluated by alkaline phosphatase (ALP) staining; adipogenic efficiency was evaluated by oil red O staining. (**H**) The differentiation rate was quantized. (**I**) Wound healing assay was performed for early and late passaged UCMSCs and (**J**) migrated cell numbers were quantified in 0 hours, 6 hours, 12 hours and 24 hours (*n* = 3). ^***^p < 0.001.

Long-term expansion of UCMSCs resulted in increased population double time (PDT) (Figure 1A), indicating the growth arrest of long-term cultured UCMSCs. Late passaged UCMSCs also showed a larger cell size than early passaged UCMSCs (Figure 1B). To describe the physiological changes of UCMSCs upon prolonged culture, we first detected the immunophenotypes expression of early passaged (P3-P6) and late passaged (P11-P14) UCMSCs. Early passaged UCMSCs expressed the typical immunophenotypes of MSCs, which showed negative of CD34, CD45, HLA-DR and positive of CD29, CD73, CD90, and CD105. Comparatively, all negative markers, especially CD45 and HLA-DR were up-regulated in late-passaged UCMSCs. A slight down-regulation of the positive marker CD105 (from 100% decreased to 86.2%), as well as an enhanced expression of CD90 (from 92.7% increased to 100%) in late passaged UCMSCs were also detected (Figure 1C). Despite these changes, the majority of the late-passaged UCMSCs maintained expression of the typical MSC markers. Therefore, spontaneous differentiation was not the most pressing factor that influenced UCMSC’s function.

We then detected expressions of senescence-associated markers. Early passaged UCMSCs showed a low positive rate (5%) of SA-β-gal stain, while more than half of the late passaged UCMSCs showed positive SA-β-gal stain (Figure 1D, 1E). The other senescence-associated marker, P16 also showed up-regulated upon passages (Figure 1F).

To evaluate the functions of UCMSCs, we analyzed the differentiation potential of UCMSCs by inducing them to osteogenic or adipogenic differentiation. The late-passaged UCMSCs showed remarkably reduced rates of osteogenic and adipogenic differentiation than the early-passaged UCMSCs. In the case of adipogenic differentiation, a massive of late-passaged cells lost during the differentiation process. This result implied that the majority of the late-passaged UCMSCs lost their differentiation potentials (Figure 1G, which was quantized in Figure 1H). We also performed a wound-healing assay. The early-passaged UCMSCs showed higher migration abilities than the late-passaged UCMSCs (Figure 1I, 1J).

Collectively, these results showed that the long-term culture-induced senescence of UCMSCs, which impaired their biological functions.

### CTDSPL up-regulation induced premature senescence of mesenchymal stem cells

**Figure 2:**
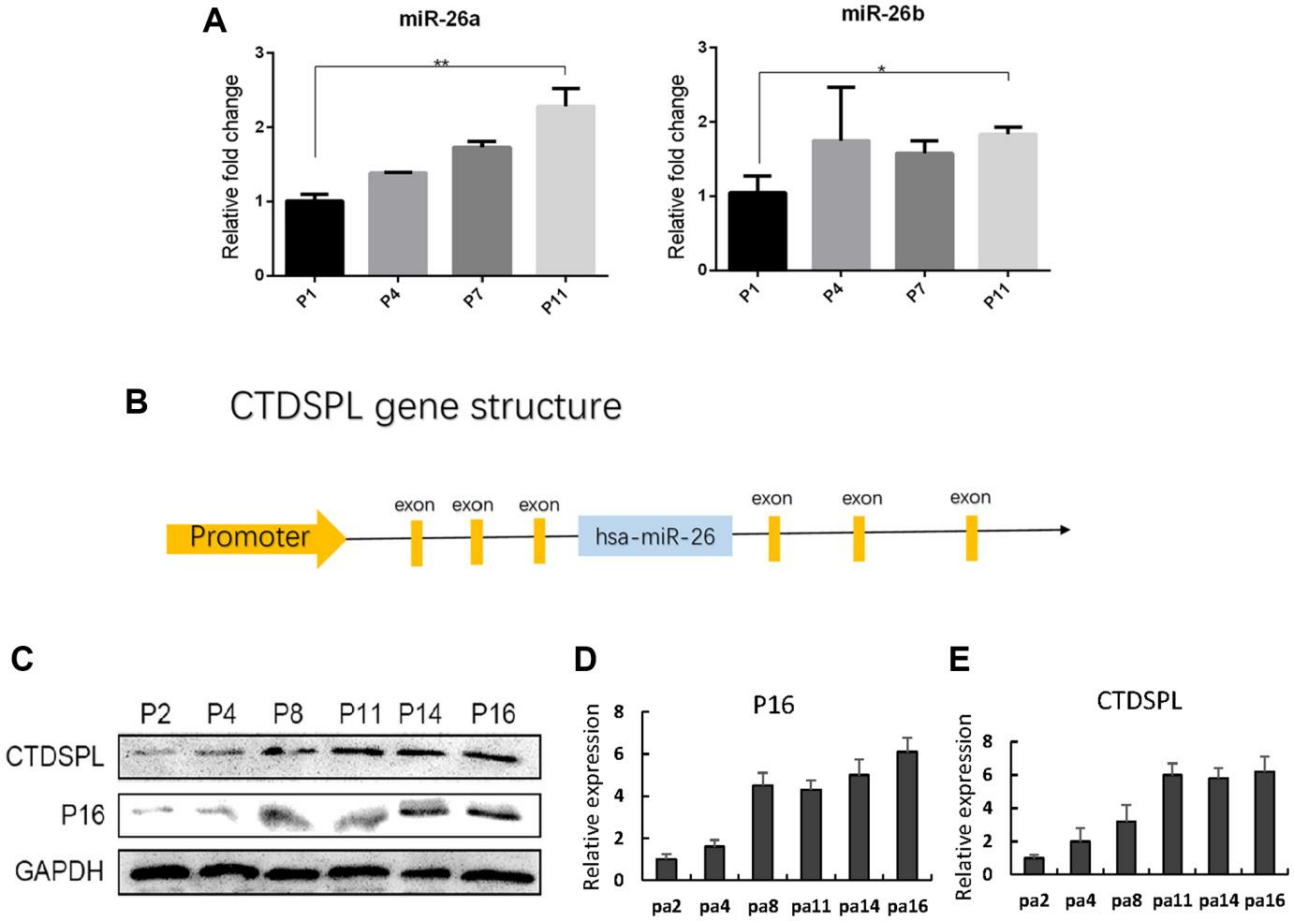
**miR-26a/b and CTDSPL expression were up-regulated following senescence of UCMSCs.** (**A**) Quantitative RT-PCR assay was performed to detect miR-26a and miR-26b expression in UCMSCs. (**B**) Gene structure of CTDSPL. (**C**) Western blot assay was performed to detect CTDSPL expression in UCMSCs. (**D**) The expression of P16 was quantized. (**E**) CTDSPL was also quantized. ^*^p < 0.05; ^**^p < 0.01.

The up-regulation of miR-26a and miR-26b in late-passaged UCMSCs has been identified in our previous study [20]. Here we first verified the expression of miR-26a/b in UCMSCs. Significant up-regulation of both miR-26a and miR-26b in long-term cultured UCMSCs (Figure 2A) was verified by quantitative RT-PCR assay. The genomic loci of miR-26a/b harbor the intron site of the CTDSP family genes (CTDSP1, CTDSP2, CTDSPL) and the expression of miR-26a/b has been closely linked to that of their host genes (Figure 2B). Therefore, the up-regulation of miR-26a/b in old UCMSCs inspired us to analyze whether their host gene CTDSPL, which was previously identified as a tumor-suppressor gene, was up-regulated in the late passage of UCMSCs. Consistent with our hypothesis, the Western blot assay showed an up-regulation of CTDSPL in late-passaged UCMSCs (Figure 2C). CTDSPL and P16 expression results were further quantized in Figure 2D, 2E.

**Figure 3:**
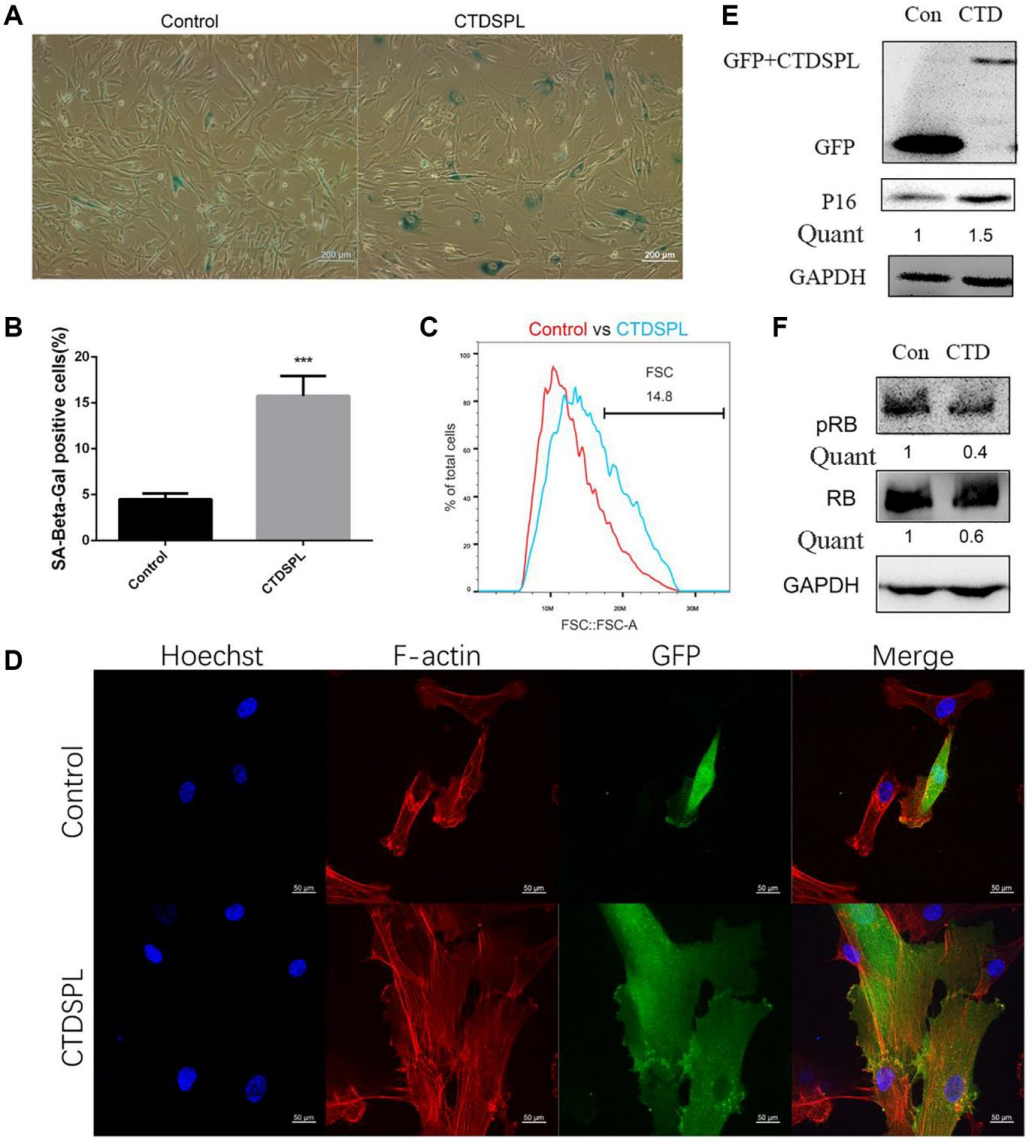
**Over-expression of CTDSPL induced premature senescence of UCMSCs.** (**A**) SA-β-gal staining of control and CTDSPL over-expressed UCMSCs. (**B**) SA-β-gal positive cells were quantified (*n* = 3). (**C**) Morphology changes were quantified by flow cytometry. (**D**) Representative confocal microscopy images of GFP tagged control or CTDSPL transfected UCMSCs. F-actin was labeled with Alexa Fluor 633 conjugated phalloidin; nuclei were stained with Hoechst 33342. (**E**) Western blot assay of p16 expression after transfected with control or CTDSPL plasmid. (**F**) Western blot assay of RB and pRB expression after transfected with control or CTDSPL plasmid. ^***^p < 0.001.

To determine whether the up-regulation of CTDSPL correlated with the senescence of MSCs, we performed a CTDSPL over-expression assay. The complete coding sequence of CTDSPL was inserted into the downstream of the GFP fluorescent protein sequence in the pEGFP-C1 plasmid. Compared to the control group, ectopic expression of CTDSPL significantly increased the rate of SA-β-gal positive cells in UCMSCs (Figure 3A, 3B). By fluorescence, we also observed that over-expression of CTDSPL resulted in an enlarged morphology change of UCMSCs (Figure 3C). As revealed by F-actin labeling (Figure 3D), this morphology change was associated with the dramatic reorganization of cytoskeletal networks. Western blot assay further showed that over-expression of CTDSPL induced the up-regulation of p16 (Figure 3E) and reduced the total and phosphorylated RB proteins (Figure 3F). Collectively, these results indicated that CTDSPL is capable of inducing senescence in UCMSCs.

### Internal microRNA-18a inhibits the senescence pathway in mesenchymal stem cells by directly repressing the expression of CTDSPL

**Figure 4:**
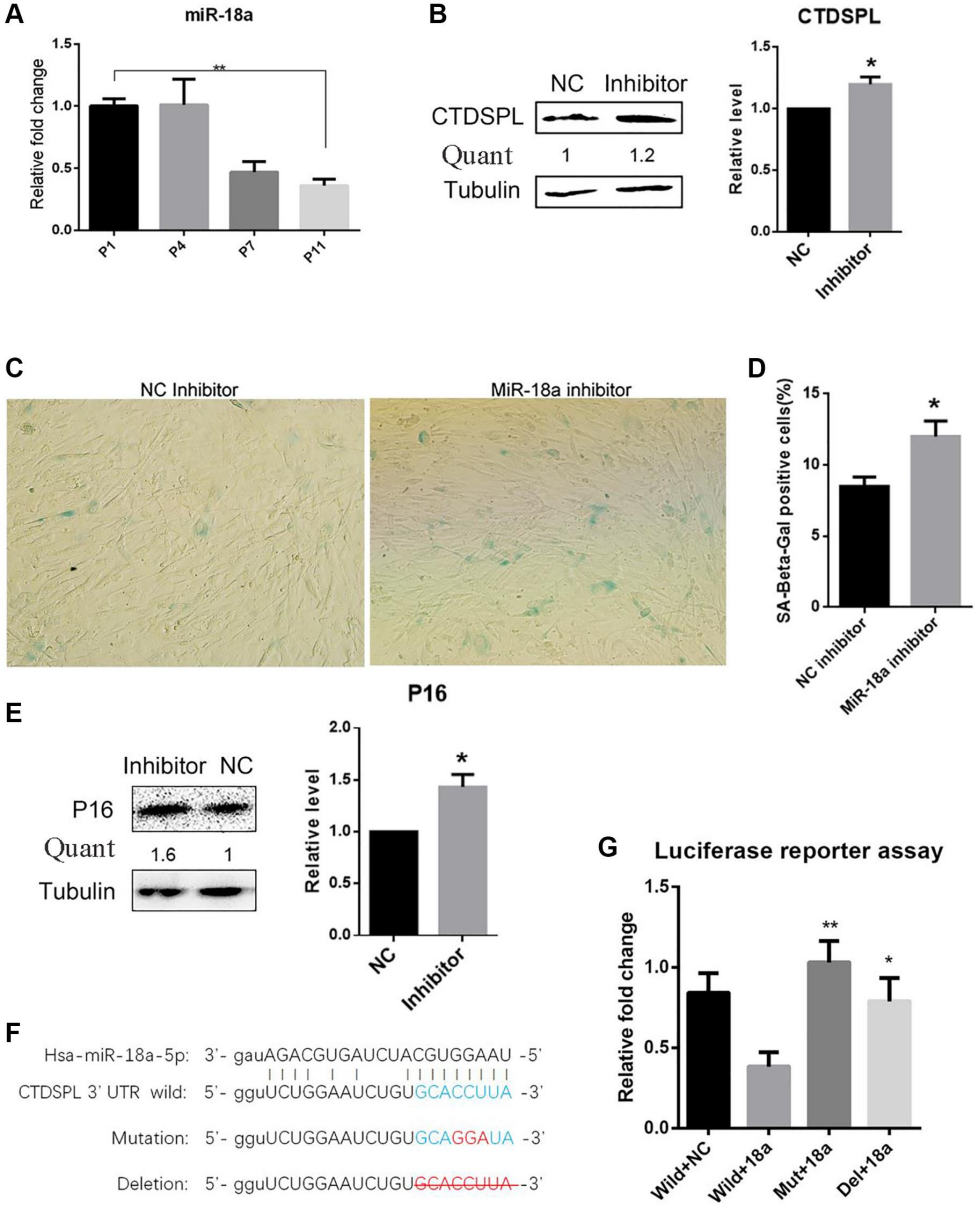
**Inhibition of miR-18a-5p induced premature senescence of UCMSCs.** (**A**) Relative expression of miR-18a-5p following passaging was analyzed by quantitate RT-PCR. (**B**) Western blot assay of CTDSPL expression after miR-NC inhibitor or miR-18a-5p inhibitor transfection. (**C**) SA-β-gal staining of UCMSCs transfected with miR-NC inhibitor or miR-18a-5p inhibitor. (**D**) SA-β-gal positive cells were quantified (*n* = 3). (**E**) Western blot assay of p16 expression in UCMSCs after miR-NC or miR-18a-5p inhibitor transfection. (**F**) Schematic representation of the reporter plasmids psiCHECK2-CTDSPL-3UTR-Wild, psiCHECK2-CTDSPL-3UTR-Mutation and psiCHECK2-CTDSPL-3UTR-Delation. (**G**) Luciferase reporter assay was performed to verify the direct repression of CTDSPL by miR-18a-5p. ^*^p < 0.05; ^**^p < 0.01.

Next, we further investigated whether CTDPL expression inhibition may repress the senescence process during long-term culture of MSCs. First, we screen the internal miRNAs that may regulate the expression of CTDSPL. The putative miRNAs that target CTDSPL were analyzed through the public miRNAs-targets database miRanda [21], Pictar2 [22], and TargetScan [23]. Among them, miR-18a-5p has been selected with the consideration of abundant expression in early-passaged UCMSCs and significant down-regulation in late-passaged UCMSCs, as shown in our previous study. To further verify this result, we measured the expression level of miR-18a-5p in different passaged UCMSCs by quantitative RT-PCR. Compared to early passaged UCMSCs, the prolonged culture of UCMSCs significantly down-regulated miR-18a-5p (Figure 4A). Furthermore, when transfected with a synthetic miR-18a-5p inhibitor, the expression of CTDSPL in UCMSCs was significantly up-regulated compared to the miR-NC control group (Figure 4B). Meanwhile, compared to UCMSCs transfected with NC inhibitor, UCMSCs transfected with hsa-miR-18a-5p inhibitor showed a larger proportion of SA-β-gal positive cells (Figure 4C, 4D) and an up-regulation of P16 expression (Figure 4E). These data suggested that hsa-miR-18a-5p played an important role in preventing the senescence of UCMSCs.

Finally, a luciferase reporter assay was performed to confirm whether the regulation effect of miR-18a-5p on CTDSPL by direct targeting mechanism. The 3′ untranslated region (UTR) sequence that included the putative miR-18a-5p binding site was inserted into the psiCheck2 dual luciferase reporter plasmid. We also constructed a mutation and a deletion plasmid as control (Figure 4F). Compared to co-transfecting miR-NC with the wide-type plasmid, the relative luciferase intensity was significantly repressed when co-transfecting miR-18a-5p with the wild-type plasmid, while co-transfecting miR-18a-5p with the mutation or deletion plasmid reversed this effect (Figure 4G). These results implied that miR-18a-5p repressed the expression of CTDSPL by directly targeting its 3′UTR site.

### Stable expression of microRNA-18a-5p attenuated senescence and improved self-renewal of mesenchymal stem cells

**Figure 5:**
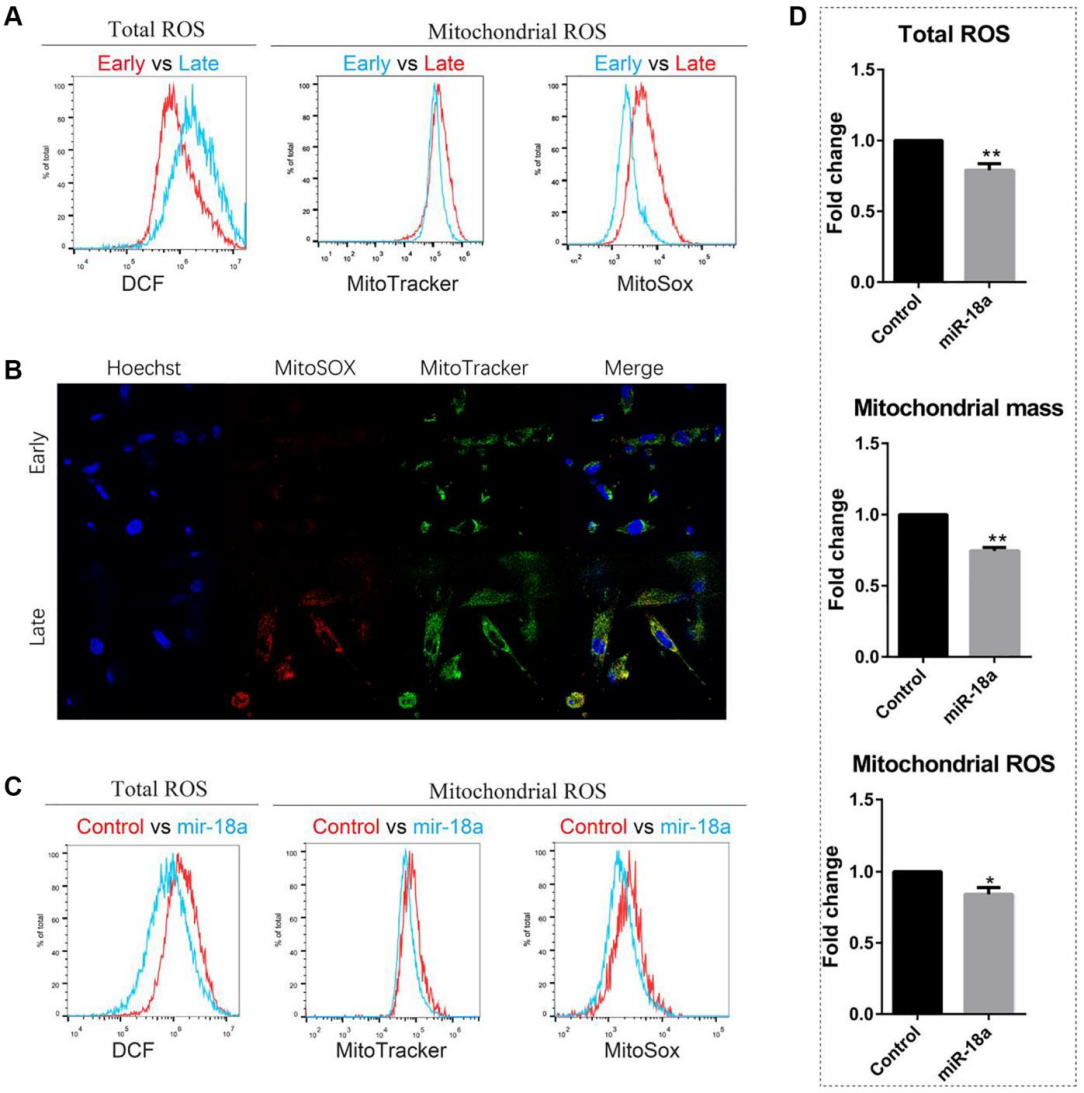
**miR-18a-5p overexpression reduced ROS levels of late passaged UCMSCs (passage 11).** (**A**, **B**) Total ROS, mitochondrial mass and mitochondrial ROS levels of early and late passaged UCMSCs were detected by flow cytometry (**A**) and confocal microscope (**B**). (**C**) Flow cytometry analysis of the total ROS, mitochondrial mass and mitochondrial ROS levels of miR-18a-5p and control lentivirus vector transduced UCMSCs and (**D**) relative fluorescence intensity of miR-18a-5p overexpressing groups relative to control groups were quantified (*n* = 3). ^*^p < 0.05; ^**^p < 0.01.

To evaluate whether continuous overexpression of miR-18a-5p could attenuate senescence and improve self-renewal of MSCs, we over-expressed miR-18a-5p by lentivirus. Since the accumulation of reactive oxygen species (ROS) impaired homeostasis and is taken as the first inducer of senescence, we used a florigenic dye DCF-DA to measure the total ROS activity within the cell. The diffused DCF-DA is first deacetylated by cellular esterases to a non-fluorescent compound, and then can be oxidized by ROS into DCF, which is a highly fluorescent compound. We found the total ROS level in the late passaged (passage 11) UCMSCs was significantly higher than the early passaged (passage 4) UCMSCs (Figure 5A). Considering mitochondria are the main source of cellular ROS, we then evaluated the mitochondrial ROS level in UCMSCs. Using the MitoTracker Green FM probe to label the mitochondria, we found the mitochondria mass increased with the senescence of UCMSCs. We then detected the mitochondrial ROS level by using the MitoSOX Red probe, which can be oxidized by mitochondrial superoxide resulting in the emission of red fluorescence. The late-passaged UCMSCs showed a higher level of mitochondrial ROS than the early-passaged UCMSCs (Figure 5A, 5B). Therefore, the senescence of UCMSCs is accompanied by an increased level of cellular ROS. However, 48 hours after overexpressing miR-18a-5p in UCMSCs, we detected reduced levels of total ROS, mitochondrial mass and mitochondrial ROS (Figure 5C, 5D). These results implied that increased expression of miR-18a-5p attenuated the senescence of UCMSCs.

**Figure 6:**
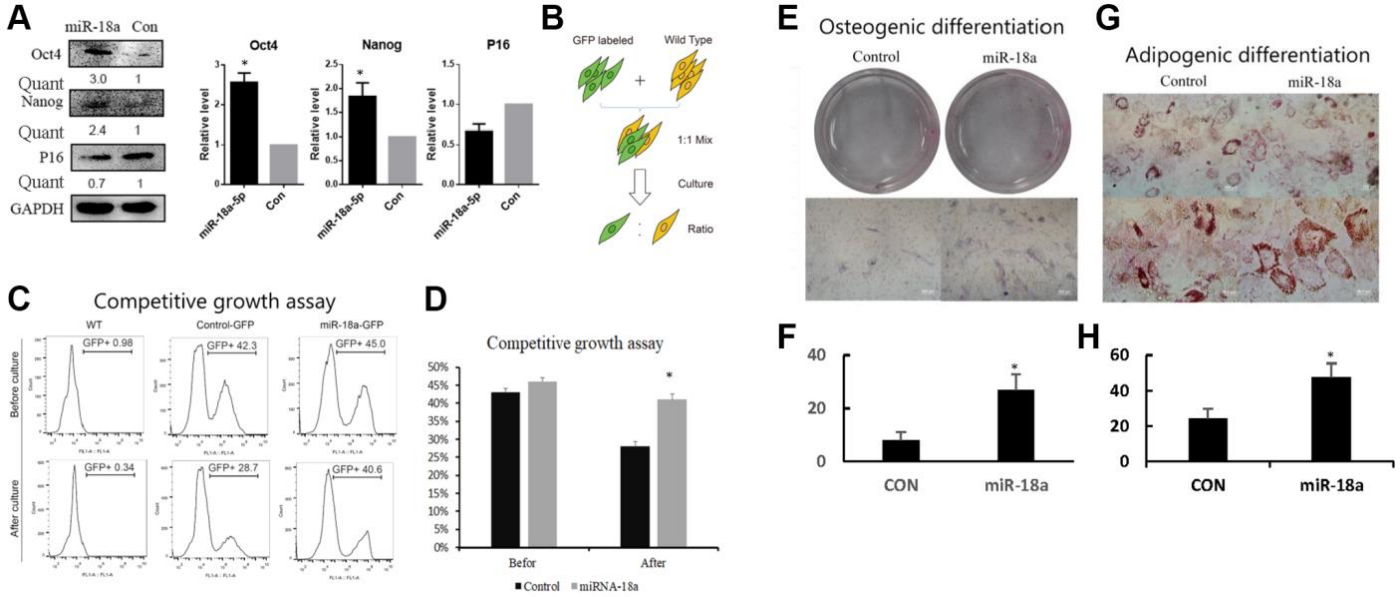
**Stable expression of miR-18a-5p improved self-renewal of UCMSCs.** (**A**) Western blot assay of Oct4, Nanog and P16 in miR-18a-5p and control lentivirus vector transduced UCMSCs. (**B**, **C**) Competitive growth assay of miR-18a-5p and control transduced UCMSCs. GFP ratios were measured by flow cytometry before and after two passaging cultures. (**D**) GFP ratio of UCMSCs in competitive growth assay before and after culture. The efficiency of osteogenic differentiation (**E**) was increased in UCMSCs stably expressing miR-18a-5p (**F**), and similarly, the potential for adipogenic differentiation (**G**) was enhanced in the group with stable miR-18a-5p transduction. Osteogenic efficiency was evaluated by ALP staining; adipogenic efficiency was evaluated by oil red O staining. The results were quantized in Figure F and Figure H. ^*^p < 0.05; ^**^p < 0.05.

To investigate whether increased expression of miR-18a-5p contributed to the self-renewal of UCMSCs, we detected the expression of Oct4 and Nanog, which are well-known stem cell markers that control self-renewal. Western blot showed that 48 hours after overexpressing miR-18a-5p, the expression of Oct4 and Nanog was up-regulated while the expression of P16 was down-regulated (Figure 6A).

To measure the effect of continuous overexpression of miR-18a-5p on cell growth, we performed a competitive cell growth assay. The GFP labeled miR-18a-5p or control lentivirus vector transduced UCMSCs were mixed with untransduced UCMSCs with a 1:1 ratio. Then the mixed cells were cultured for another two passages and the GFP+/GFP- ratio was measured by flow cytometry (Figure 6B). Before passaging, the percentage of GFP+ cells were 42.3% for control and 45.0% for miR-18a-5p over-expressing cells. After two passages, the percentage of GFP+ cells in the control group dropped to 28.7%, while the percentage of GFP+ cells expressing miR-18a-5p maintained a similar ratio (40.6%) (Figure 6C, 6D). These results indicated that stable expression of miR-18a-5p improved the growth activity of UCMSCs.

Finally, to evaluate the differentiation potentials of UCMSCs stably expressing miR-18a-5p, we performed an osteogenic and adipogenic differentiation assay. The results showed that stable transduction of miR-18a-5p enhanced both the osteogenic and adipogenic differentiation ability of UCMSCs (Figure 6E, 6G and quantized in Figure 6F, 6H respectively).

## DISCUSSION

Here we demonstrated that miR-18a-5p played a pivotal role in preventing senescence and maintaining self-renewal of MSCs. We found CTDSPL was a potent inducer of senescence in MSCs. The up-regulation of CTDSPL accompanying with miR-26a/b expression was detected in the long-term cultured UCMSCs. Ectopic expression of CTDSPL induced a significant change in cell morphology and positive expression of other senescence-related markers. We identified miR-18a-5p as a repressor of CTDSPL. Reduced expression of miR-18a-5p resulted in the up-regulation of CTDSPL and further premature senescence of MSCs. However, continuous overexpression of miR-18a-5p attenuated senescence and improved self-renewal of MSCs.

*Ex vivo* expanded MSCs underwent progressive biological changes. Previous studies reported a decreased expression of typical MSC immunophenotypes and an aberrant expression of development and lineage-specific genes in long-term cultured MSCs, which suggested a trend of spontaneous differentiation [24]. In our data, the immunophenotype changes between early and late passaged UCMSCs did not imply significant spontaneous differentiation. Although the lack of MSC-specific markers made it hard to monitor the purity of MSCs, the majority of the cultured populations still kept their typical phenotypes during the culture. Due to the lack of telomerase activity, replicative MSCs will evolve toward a state of cell cycle arrest [25, 26]. We detected the up-regulation of P16 and accumulation of SA-β-gal in the late-passaged UCMSC, which implied a senescent state of the MSCs. In line with previous studies, long-term cultured UCMSCs exhibited impaired differentiation potentials and migration properties [27, 28].

In this work, UCMSCs were isolated and cultured as previously described, and confirmed to meet the criteria established by the International Society for Cellular Therapy (ISCT) for defining MSCs [29]. It has been demonstrated that following ISCT guidelines for MSC isolation leads to the establishment of heterogeneous, non-clonal cultures of stromal cells, containing stem cells with varying multipotential properties, committed progenitors, and differentiated cells [30, 31]. Therefore, further research should be conducted utilizing more purified cell populations or single-cell level studies.

Along with the senescence of UCMSCs, we detected a significant up-regulation of miR-26a/b and its host gene, CTDSPL. As an important tumor-suppressor gene, over-expression of miR-26a/b was reported to initiate apoptosis or arrest cell cycle [32]. As located on the intron sites of CTDSP1/2/L family genes, the expression of miR-26a/b is closely correlated with the expression of CTDSP1/2/L. Particularly, a previous study showed a cooperative function of miR-26a/b with its host genes [16]. The CTDSP1/2/L family was first reported by Michele to regulate transcription by catalyzing the dephosphorylation of RNA polymerase II [33]. They were also taken as a critical repressor of neuronal genes in global tissues [34]. Other studies also reported the involvement of the regulation of TGFβ/BMP activities and snail protein stability by members of the CTDSP family [35, 36]. Nevertheless, each functional role of the CTDSP family genes in different cells is still under investigation. This study focused on the regulation of CTDSPL in UCMSCs. Owing to the frequent mutation, the expression of CTDSPL has been impaired in many epithelial tumors, indicating a tumor-suppressor role of CTDSPL [18]. Considering that senescence is an important anti-tumor mechanism in mammalian cells, it was not surprising to find that the up-regulation of CTDSPL significantly induced senescence of UCMSCs. In our study, over-expression of CTDSPL both reduced the phosphorylated and total RB expression, which suggested a cell cycle arrest of MSCs. It was reported that CTDSPL arrested the cell cycle by dephosphorylating pRB [18] and a down-regulation of total RB expression has been described in senescent MSCs^34^. Nevertheless, more evidence still needs to verify the direct regulation of the cell cycle by CTDSPL. It would be also interesting to see whether other activities were involved in CTDSPL to the senescence of UCMSCs.

We showed that the maintenance of miR-18a-5p played an important role in preventing senescence of UCMSCs. MiR-18a-5p belongs to the miR-17/92 cluster (miR-17, miR-18a, miR-19a, miR-19b-1, miR-20a, miR-92a-1). Known as oncogenes, the expression of miR17/92 cluster plays critical roles in regulating cell proliferation and apoptosis in cancer cells. As to the normal tissues, abundant expression of the miR-17/92 cluster was detected in the lung, heart, B cells and many other tissues. Loss-of-function of miR-17/92 cluster resulted in severe developmental defects of these tissues [37]. These results suggested that the miR-17/92 cluster plays a pivotal role in regulating fundamental processes related to development. The regulation of aging or senescence is also involved. The down-regulation of members of the miR-17/92 cluster have been discovered in many aged tissue types [38]. Overexpression of miR-17 was previously found to be able to restore the therapeutic potential of old MSCs [23]. Consistent with these results, senescent MSCs derived from human umbilical cord and umbilical cord blood also showed down-regulations of members of the miR-17/92 cluster. Compared to its other members, miR-18a-5p shows a different seed sequence, which suggests a distinct functional role. By a combination of the three miRNA-targets databases, miRanda, Pictar2, and TargetScan, we identified miR-18a-5p as a putative mediator of CTDSPL. In comparison, the other members, miR-17, miR-20a or miR-92a only showed a weak binding score or failed to be predicted by the databases. By a luciferase reporter assay, we confirmed the direct repression of CTDSPL by miR-18a-5p. Furthermore, we demonstrated that the inhibition of miR-18a-5p promoted the expression of CTDSPL as well as induced senescence of UCMSCs. Continuous overexpression of miR-18a-5p reduced the cellular ROS level, promoted the expression of stem cell typical genes, and improved the growth and differentiation potentials of MSCs. These results highlighted the importance of miR-18a-5p to the self-renewal and maintenance of MSCs. The expression of miR-17/92 cluster, which includes miR-18a-5p, has been demonstrated to be promoted by MYC [39, 40]. Given that MYC is an important regulator of the self-renewal of stem cells, it could be suggested that MYC through miR-18a-5p suppressed the expression of CTDSPL in UCMSCs. Interestingly, it was also reported that MYC could repress miR-26a expression, which could negatively regulate MYC expression through the repression of EZH2 expression [41, 42].

There are still many limitations in this study that need to be addressed in future work. For example, the Retinoblastoma (Rb) family, RB1, RB2/P130, and P107, which regulate multiple aspects of cellular behavior such as cell cycle progression, senescence, apoptosis, and differentiation. Murine studies have suggested functional redundancy among Rb family proteins, while initial knockout analyses indicated an ancillary role for Rb2/p130 and P107 [43]. However, more recent research has demonstrated that RB2 alone plays a crucial role in inducing senescence [44], which should not be overlooked. Moreover, several studies have demonstrated that other factors besides cell senescence can cause an increase in SA-β-gal activity, making this enzyme unsuitable for age-related assessments [45]. Additionally, cellular senescence should be evaluated through Western blotting analyses of further proteins involved in senescence and cell cycle exit, such as RB, RB2, p107, and p27 [46, 47], in addition to the already assessed p21 and p53. The use of senescence-associated β-galactosidase (SA-β-gal) activity as a marker for cellular senescence is debatable due to its lack of specificity. Cell migration assay may only measure total cell counts within the exclusion zone, therefore confounding results on the contribution of proliferation versus migration.

In this study, we identified that prolonged *in vitro* culture increased CTDSPL expression, which attenuated proliferation and promoted senescence of UCMSCs. We demonstrated that miR-18a-5p was an important repressor of CTDSPL. Stable overexpression of miR-18a-5p prevents senescence and maintains self-renewal of UCMSCs. Therefore, our work provided a promising strategy to maintain the therapeutic functions of MSCs. Considering the close links of miR-18a-5p, CTDSPL and senescence of MSCs, the expression of miR-18a-5p and CTDSPL may also serve as useful biomarkers to monitor therapeutic activities of MSCs during *ex vivo* expansion.

## METHODS

### Cell culture

Human umbilical cord tissue slices were kindly donated from Shandong Cell-Tissue Bank (Jinan, Shandong, China). The isolation of UCMSCs was performed as previously described [15]. Briefly, arteries and veins were first removed and the remaining tissues were chopped into small pieces. All pieces were placed in a DMEM-low glucose medium supplemented with 5% fetal bovine serum (Hyclone, South Logan, UT, USA) and 10 ng/ml basic fibroblast growth factor (Peprotech, London, UK) and cultured in 37°C at 5% CO_2_. In total, UCMSCs from five samples were used in this study. HEK293T cells were purchased from Applied Biological Materials China (Nanjing, Jiangsu, China) and maintained with DMEM-high glucose medium supplemented with 10% fetal calf serum (Hyclone) in 37°C at 5% CO_2_.

### Osteogenic and adipogenic differentiation

For osteogenic differentiation, confluent UCMSCs growing in six-well plates were subjected to osteogenic medium composed of DMEM with low glucose supplemented with 10% FBS (Hyclone), 1% penicillin/streptomycin (Hyclone), 100 nM dexamethasone (Sangon Biotech, Shanghai, China), 10 mM β-glycerophosphate (Sigma, St. Louis, MO, USA) and 50 μM ascorbic acid (Sigma). At the end of the three weeks, cells were fixed with 4% paraformaldehyde for 30 minutes at room temperature and stained with BCIP/NBT (Amresco, Solon, OH, USA) for the visualization of alkaline phosphatase (ALP) expression. For adipogenic differentiation, confluent UCMSCs growing in six-well plates were treated with DMEM with low glucose supplemented with 10% fetal bovine serum (Hyclone), 1% penicillin/streptomycin (Hyclone), 1 μM dexamethasone (Sangon Biotech), 500 μM isobutylmethylxanthine (Sigma), 200 μM indomethacin (Sigma) and 10 μg/ml insulin (BasalMedia, Shanghai, China). At the end of the three weeks, cells were fixed with 10% buffered formalin for 30 minutes at room temperature and stained with oil red O for the visualization of oil droplets.

### Plasmids construction

For the CTDSPL overexpression plasmid, the coding sequence of CTDSPL was inserted between the HindIII and BamHI sites in the pEGFP-C1 plasmid. For the wild-type luciferase reporter plasmid, the part sequence of CTDSPL 3′ untranslated region (UTR) that contains the putative miR-18a-5p binding site was inserted between the XhoI and NotI sites in psiCHEKC2 vector (Promega, Madison, WI, USA). For the 3′UTR mutated and deleted reporter plasmids, the mutation or deletion of specific sites in wild-type reporter plasmid was generated using a Mut Express II Fast Mutagenesis Kit (Vazyme, Nanjing, Jiangsu, China) according to the manufacturer’s procedure. To construct the lentivirus transduced miR-18a-5p overexpression plasmid, the primary miR-18a-5p sequence was inserted between the BamHI and XbaI sites in the pLVX-AcGFP-N1 vector. To construct a GFP-labeled lentivirus transduced miR-18a-5p overexpression plasmid, the primary miR-18a-5p sequence was inserted into the XbaI site in the pLVX-AcGFP-N1 vector.

### Cell transfection

To overexpress CTDSPL in MSCs, the Neon electroporation system (Invitrogen, Eugene, OR, USA) was used according to the manufacturer’s guidelines. The empty pEGFP-C1 plasmid was used as a control. The transfected MSCs were seeded into six-well plates and cultured for 72 hours. To inhibit miR-18a-5p in MSCs, the synthetic miR-18a-5p inhibitors (GenePharma, Shanghai, China) were transfected into MSCs using a Neon electroporation system (Invitrogen). The electroporation condition was 1350V and one pulse with pulse width 30 ms. The transfected cells were cultured for 72 hours. The non-sense inhibitors served as a control. For the luciferase reporter assay, HEK293T cells were seeded into 96-well plates and cultured until 80% confluence. The synthetic miR-18a-5p mimics (GenePharma) and luciferase reporter plasmids were co-transfected into HEK293T cells using Lipofectamine 2000 (Invitrogen) following the manufacturer’s procedure.

### Immunophenotypes assay

To characterize the immunophenotypes of UCMSCs, early passaged (passage 3 to passage 6) or late passaged (passage 11 to passage 14) UCMSCs were collected and incubated with fluorescence conjugated antibodies against CD34, CD45, HLA-DR, CD73, CD105 (Miltenyi Biotec, Bergisch Gladbach, Germany), and CD29, CD90 (BD Bioscience, San Jose, CA, USA). Fluorescence intensity was detected using the Accuri C6 flow cytometer (BD Bioscience). Analysis was performed using FlowJo software (Tree Star, Inc., San Carlos, CA, USA).

### Cell migration assay

UCMSCs were cultured in six-well plates until 100% confluence. Then the cells were scratched with 100 μl pipette tips, washed with PBS and incubated for another 24 hours. Pictures were obtained under an inverted microscope (Nikon Eclipse Ti-S, Tokyo, Japan) at different time points. The results were analyzed using ImageJ software (developed at the National Institutes of Health).

### Quantitative RT-PCR assay

A stem-loop RT-PCR method was used to quantify the expression of mature miRNAs [48]. Total RNAs were isolated using RNAiso plus (Takara, Dalian, Liaoning, China) following standard procedure. First-strand cDNA was generated with miRNA-specific RT primers using M-MLV reverse transcriptase (Promega, Madison, WI, USA). Quantitative RT-PCR (qRT-PCR) was performed with EvaGreen (Biotium, Hayward, CA, USA) using ABI 7500 Real-time PCR instrument (Applied Biosystems, Foster City, CA, USA). U6 was used as a housekeeping gene. And 2^−ΔΔCT^ method was used to analyze the data. The primers used in this study were as follows:

hsa-miR-26a-5p, 5′-GTCGTATCCAGTGCAGGGTCCGAGGTATTCGCACTGGATACGACagccta-3′ (RT primers), 5′-CCGCCGTTCAAGTAATCCAG-3′ (forward); has-miR-26b-5p, 5′-GTCGTATCCAGTGCAGGGTCCGAGGTATTCGCACTGGATACGACacctat-3′ (RT primers), 5′-CGCCGCTTCAAGTAATTCAGGAT-3′ (forward); hsa-miR-18a-5p, 5′-GTCGTATCCAGTGCAGGGTCCGAGGTATTCGCACTGGATACGACCTATCT-3′ (RT primers), 5′-CACGCGTAAGGTGCATCTAGT-3′ (forward); universal reverse primers, 5′-CCAGTGCAGGGTCCGAGGTA-3′; U6, 5′-CTCGCTTCGGCAGCACA-3′ (forward), 5′-AACGCTTCACGAATTTGCGT-3′ (reverse); CTDSPL, 5′-cataagcttacatggacggcccggccatcat-3′ (forward), 5′-gacggatccctacctattgcagagtctgtg-3′ (reverse).

### Western blot assay

Whole-cell lysates of MSCs were extracted using a total protein extraction kit (Sangon Biotech, Shanghai, China). Protein samples were resolved by 12% SDS-PAGE and transferred to PVDF membranes (Millipore, Billerica, MA, USA). Membranes were blocked with 5% non-fat milk or bovine serum albumin (BSA) for 1 hour at room temperature and subsequently incubated overnight at 4°C with diluted primary antibodies. The primary antibodies used in this study included: CTDSPL (Santa Cruz Biotech, Santa Cruz, CA, USA), p16 (Santa Cruz Biotech), pRB (Cell Signaling Technology, Beverly, MA, USA), RB (Cell Signaling Technology), GAPDH (Santa Cruz Biotech) and Tubulin (Transgene Biotech, Beijing, China). To detect the signals, the membranes were then incubated with the appropriate horseradish peroxidase-conjugated secondary antibody (CWBio, Beijing, China) for 1 hour at room temperature. Finally, the signals were visualized using an enhanced chemiluminescence substrate (ECL; CWBio).

### SA-β-gal activity assay

To examine senescence-associated β-galactosidase (SA-β-gal) activity, MSCs were seeded into six-well plates. Senescence-associated SA-β-gal staining was performed using an SA-β-gal staining kit (Beyotime, Shanghai, China). Cells were observed using an inverted microscope (Nikon Eclipse Ti-S). Three random fields per well were selected to count SA-β-gal positive cells.

### Cytoskeleton labeling and imaging

PEGFP-CTDSPL or empty vector-transfected MSCs were fixed with 4% paraformaldehyde (PFA) for 20 minutes, permeabilized with 0.1% Triton X-100 for 5 minutes and blocked with 1% bovine serum albumin (BSA) for one hour. Then, the cells were stained with Alexa Fluor 633 Phalloidin (Invitrogen) for half an hour and Hoechst 33342 for 10 minutes. All procedures were performed at room temperature. Fluorescence was detected using a spinning disk confocal microscope system (Andor Technology, Belfast, Northern Ireland) on an inverted microscope (Nikon Eclipse Ti-E, Tokyo, Japan).

### Lentivirus transduction

For preparation of lentivirus, 293T cells were transfected with a mixture of plasmids containing lentiviral vector (control and miR-18a-5p) and packaging plasmids (pMDL, pVSVG and pREV) using CaPO_4_ precipitation according to a previously described protocol [49]. Media containing lentiviruses were collected at 24 hours and 48 hours after transfection. Then the media were filtered through a 0.45 μm pore size filter and centrifuged at 100,000 × g for 70 min at 4°C. After pouring off the supernatant, the pellet was resuspended in 100 μl PBS and stored at −80°C. For the transduction of MSCs, cells were seeded in a six-well plate. The next day, lentiviral supernatants supplemented with 10 μg/ml polybrene (Sigma) were added. Cells were incubated for another 24 hours before changing to fresh media.

### Reactive oxygen species assay

MSCs cultured in six-well plates were incubated with 5 μM DCFH-DA (Beyotime) for 20 minutes, or 100 nM MitoTracker Green FM (Invitrogen) for 15 minutes, or 5 μM MitoSOX Red (Invitrogen) for 10 min at 37°C protected from light. For flow cytometry assay, cells were washed with fresh media three times, and detached with trypsin. For confocal microscope imaging, cells were counter-stained with Hoechst33342 to visualize the nuclei.

### Luciferase reporter assay

To test the binding of miR-18a-5p to CTDSPL, HEK293T cells were pre-seeded into a 96-well plate one day before transfection. Then the cells were co-transfected with: 1) non-sense oligonucleotides control and wild type CTDSPL-3′UTR plasmid; 2) miR-18a-5p mimics and wild type CTDSPL-3′UTR plasmid; 3) miR-18a-5p mimics and mutated CTDSPL-3′UTR plasmid; 4) miR-18a-5p mimics and deleted CTDSPL-3′UTR plasmid. After 48 hours post-transfection, Renilla and firefly luciferase activities were measured using the Dual-Glo Luciferase Assay System (Promega, Madison, WI, USA) according to the manufacturer’s instructions.

### Statistical analysis

All data were presented as means ± SEM. Statistical analysis was performed using Graphpad Prism (Graphpad Software. San Diego, CA, USA). A two-tail student’s *t*-test was performed to evaluate the significance level of the two groups. A *p*-value of less than 0.05 was considered statistically significant.
